# P-1641. Risk Factors for COVID-19 Hospitalization in the Elderly Population at Maharaj Nakorn Chiang Mai Hospital

**DOI:** 10.1093/ofid/ofaf695.1817

**Published:** 2026-01-11

**Authors:** Natthapong Suthammopasut, Panas Jesadaporn, Surachet Vongsanim, Parichat Salee

**Affiliations:** Faculty of Medicine, Chiang Mai University, Chiang Mai, Chiang Mai, Thailand; Faculty of Medicine, Chiang Mai University, Chiang Mai, Chiang Mai, Thailand; Faculty of Medicine, Chiang Mai University, Chiang Mai, Chiang Mai, Thailand; Faculty of Medicine, Chiang Mai University, Chiang Mai, Chiang Mai, Thailand

## Abstract

**Background:**

COVID-19 has caused substantial morbidity and mortality worldwide, with elderly individuals disproportionately affected. However, data on hospitalization risk factors among older adults in Thailand are limited. This study aimed to characterize clinical features and identify predictors of hospitalization among elderly COVID-19 patients at Maharaj Nakorn Chiang Mai Hospital.

**Figure:**
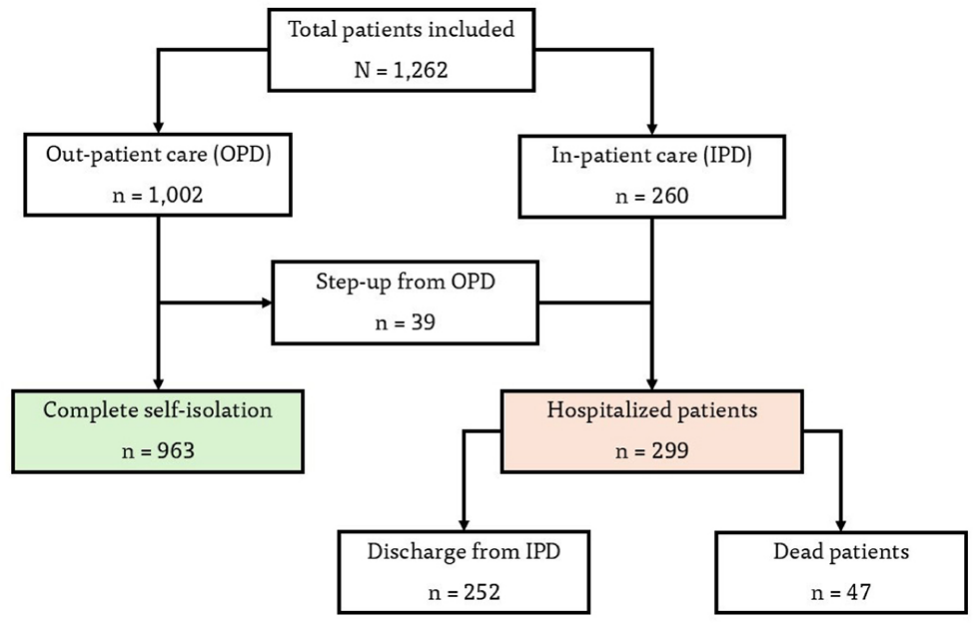
Study Flow Diagram and Distribution of Hospitalized and Non-Hospitalized Elderly Patients We included 1,262 elderly patients in the study. A total of 299 (23.7%) patients were hospitalized, including 260 (20.6%) who were primarily hospitalized at presentation, while 39 out of 1,002 patients were subsequently hospitalized after failed out-patient care. Only 47 (3.7%) died in our cohort.

**Figure:**
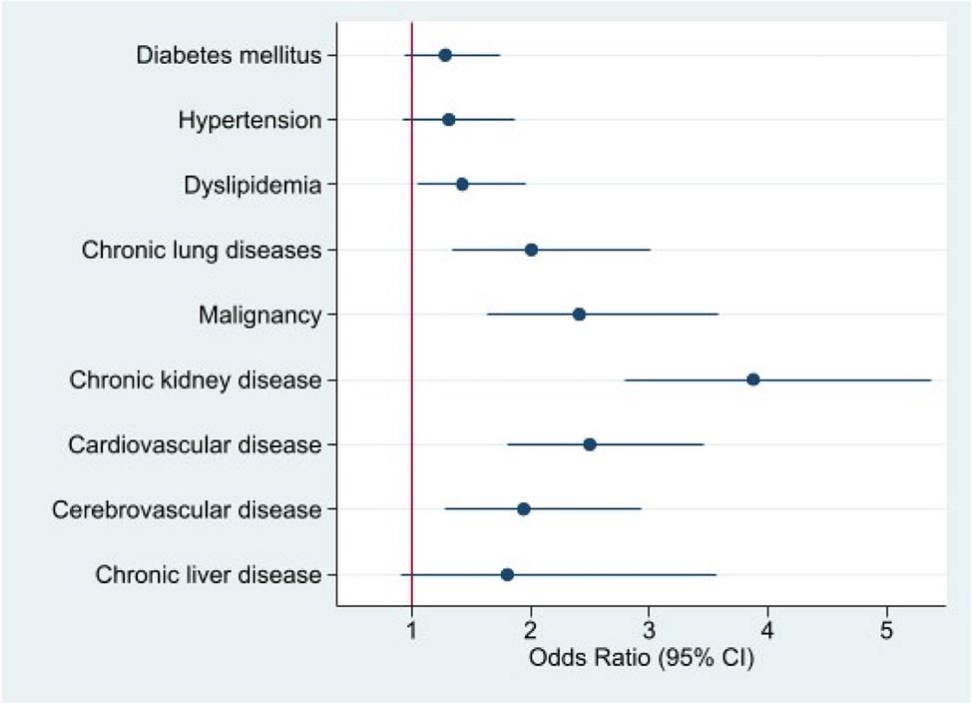
Forest Plot of Multivariate Logistic Regression: Comorbidities Associated with Hospitalization Chronic kidney disease (CKD) and cardiovascular disease were most strongly associated with hospitalization (OR 2.70 and 2.04, respectively). Other significant conditions included hypertension, diabetes, dyslipidemia, malignancy, and cerebrovascular disease. Multivariate analysis identified malignancy, CKD, cardiovascular disease, and cerebrovascular disease as independent predictors.

**Methods:**

A retrospective cohort study was conducted on 1,262 patients aged ≥60 years with confirmed SARS-CoV-2 infection between January 2021 and December 2022. Multivariate logistic regression was used to identify predictors of hospitalization, oxygen therapy, and mortality. Variables included demographics, comorbidities, vaccination status, clinical symptoms, and infection period (Delta vs. Omicron variants). A clinical prediction model was developed and validated.

**Figure:**
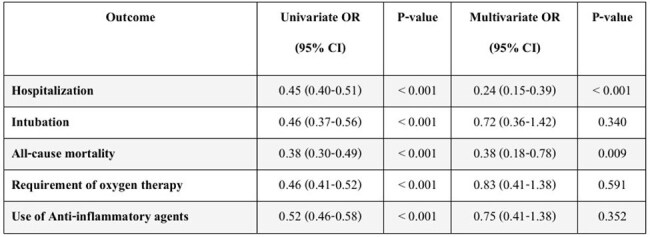
Impact of SARS-CoV-2 Vaccination on Clinical Outcomes in Elderly COVID-19 Patients Patients with ≥ 2 vaccine doses had significantly lower hospitalization rates. Logistic regression showed significant reductions in hospitalization (OR 0.45), intubation (OR 0.46), mortality (OR 0.38), oxygen use (OR 0.46), and anti-inflammatory therapy (OR 0.52) in univariate analysis. Multivariate analysis confirmed a 76% reduction in hospitalization (OR 0.24, 95% CI: 0.15–0.39) and 62% reduction in mortality (OR 0.38, 95% CI: 0.18–0.78)

**Figure:**
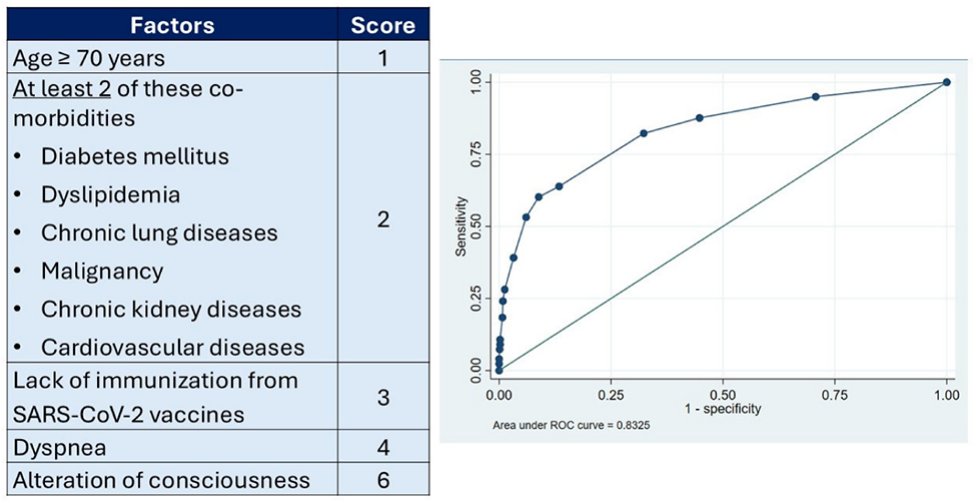
Clinical Prediction Score Components and ROC Curve for Hospitalization Risk Stratification A clinical scoring system was developed using variables with significant associations in multivariate analysis: age ≥70, comorbidities (diabetes, dyslipidemia, chronic lung disease, malignancy, CKD, cardiovascular disease), severe symptoms (dyspnea, altered consciousness), and unvaccinated status. The model performed with an AUC of 0.83, sensitivity of 82.3%, and specificity of 67.7% at a cut-off score ≥ 3. Internal validation showed 44.1% of patients scored ≥ 3, though the observed hospitalization rate was 23.7%.

**Results:**

Among all patients, 299 (23.7%) required hospitalization. Chronic kidney disease (adjusted OR 3.88, 95% CI: 2.80–5.37) and cardiovascular disease (OR 2.50, 95% CI: 1.81–3.46) were strong predictors. Vaccination significantly reduced hospitalization (OR 0.24, 95% CI: 0.15–0.39) and mortality (OR 0.38, 95% CI: 0.18–0.78). Hospitalization rates were highest during the Delta wave (51.2%) compared to Omicron BA.1/BA.2 (19.6%) and BA.4/BA.5 (26.8%, p < 0.001). Dyspnea (OR 7.27, 95% CI: 4.81-10.98) and altered consciousness (OR 27.28, 95% CI: 5.97-124.54) were the most predictive clinical features. A predictive scoring model (cut-off ≥ 3) demonstrated good performance with sensitivity of 82.3%, specificity of 67.7%, and AUC of 0.83.

**Conclusion:**

Hospitalization risk among elderly COVID-19 patients was significantly associated with comorbidities, clinical symptoms, variant type, and vaccination status. Vaccination provided strong protection against severe outcomes. The predictive score may help guide early triage and resource allocation in high-risk populations during future COVID-19 waves.

**Disclosures:**

All Authors: No reported disclosures

